# Effect of Nitrogen Addition on Selection of Germination Trait in an Alpine Meadow on the Tibet Plateau

**DOI:** 10.3389/fpls.2021.634850

**Published:** 2021-05-14

**Authors:** Kun Liu, Yang Liu, Zhilong Zhang, Shiting Zhang, Carol C. Baskin, Jerry M. Baskin, Ting Liang, Haiyan Bu, Shuxia Li, Tingting Zhang, Xianliang Cui, Sa Xiao

**Affiliations:** ^1^State Key Laboratory of Grassland and Agro-Ecosystems, School of Life Sciences, Lanzhou University, Lanzhou, China; ^2^Department of Chemistry and Life Science, Gansu Normal College for Nationalities, Hezuo, China; ^3^Department of Biology, University of Kentucky, Lexington, KY, United States; ^4^Department of Plant and Soil Sciences, University of Kentucky, Lexington, KY, United States; ^5^School of Biological and Chemical Science, Puer University, Puer, China

**Keywords:** alpine meadow, germination traits, nitrogen addition, species diversity, Tibet Plateau

## Abstract

Seed germination requirements may determine the kinds of habitat in which plants can survive. We tested the hypothesis that nitrogen (N) addition can change seed germination trait-environmental filter interactions and ultimately redistribute seed germination traits in alpine meadows. We determined the role of N addition on germination trait selection in an alpine meadow after N addition by combining a 3-year N addition experiment in an alpine meadow and laboratory germination experiments. At the species level, germination percentage, germination rate (speed) and breadth of temperature niche for germination (BTN) were positively related to survival of a species in the fertilized community. In addition, community-weighted means of germination percentage, germination rate, germination response to alternating temperature and BTN increased. However, germination response to wet-cold storage (cold stratification) and functional richness of germination traits was lower in alpine meadows with high-nitrogen addition than in those with no, low and medium N addition. Thus, N addition had a significant influence on environmental filter-germination trait interactions and generated a different set of germination traits in the alpine meadow. Further, the effect of N addition on germination trait selection by environmental filters was amount-dependent. Low and medium levels of N addition had less effect on redistribution of germination traits than the high level.

## Introduction

Trait-based community ecology is a new approach to understanding community assembly (Díaz et al., 2004; McGill et al., 2006). Functional traits have been considered as common currency to predict the performance of species, species composition of communities and how these things shift over time and space (Larson and Funk, 2016). A trait–filter community assembly theory has been developed and applied to help understand how environmental factors and plant traits act jointly to determine community structure (Cornwell and Ackerly, 2009; Larson and Funk, 2016). Thus, analyses of functional traits have become fundamental tools for predicting the responses of a plant community to environmental change. Although the number of studies on plant functional traits and community assembly is increasing, most of them have focused on easily measured traits (e.g., plant height, specific leaf area, and seed mass) and only a few on seed germination traits (Jiménez-Alfaro et al., 2016; Liu et al., 2018, 2020; Larson et al., 2020).

Seed germination is the earliest expressed life history trait (Donohue et al., 2010), and it can directly or indirectly affect the expression of post-germination traits that are closely related to plant fitness (Simons and Johnston, 2000; Donohue, 2002). Therefore, seed germination can potentially influence the composition of plant communities (Wu and Du, 2007; Saatkamp et al., 2019). Many factors such as light, temperature and soil moisture can affect seed dormancy-breaking and germination (Fenner and Thompson, 2005; Bewley et al., 2013; Baskin and Baskin, 2014). Generally, these factors act together to determine the timing of seed germination and thus play an important role in seedling survival (Westoby, 1981; Densmore and Zasada, 1983; Baskin and Baskin, 2014). The breadth of the temperature niche for germination and how it changes are important determinants of the seed germination season of plants in regions with seasonal temperature changes (Baskin and Baskin, 2014). For example, a wet-cold storage requirement for dormancy-break can prevent seeds from germinating soon after dispersal in autumn, thus avoiding seedling death from freezing in winter (Liu et al., 2011). Further, a fluctuating temperature requirement for germination restricts germination to vegetation gaps, thus avoiding seedling death due to shading by neighboring plants (Thompson et al., 1977; Liu et al., 2013). To some extent, environmental requirements for seed germination of a plant species are important determinants for its existence in a given community and environment (Fenner and Thompson, 2005; Larson and Funk, 2016).

Soil nitrogen (N) is an important factor that can affect plant fitness and interspecific interactions (Hautier et al., 2009; Milbau et al., 2017; Hämmerle et al., 2018). For example, N addition can increase the intensity of competition for light and result in loss of some species in grassland communities (Hautier et al., 2009). Thus, N is an important environmental filter in determining community structure and ecosystem function. Due to human activities, the concentration of atmospheric N and N deposition are increasing (Gruber and Galloway, 2008; Ackerman et al., 2019). The Tibet Plateau is experiencing serious N deposition. For example, data collected from 1990 to 2003 show a mean of 7.55 kg ha^–1^ year^–1^, a minimum of 1.08 kg ha^–1^ year^–1^ and a maximum level of 17.81 kg ha^–1^ year^–1^ of N deposition in Qinghai (on the Tibet Plateau) (Lü and Tian, 2007). It previously has been shown that this level of N deposition in Qinghai can change the species composition of alpine meadows on the Tibet Plateau (Zong et al., 2016).

With increased atmospheric N deposition and increased fertilizer use in agriculture becoming global problems, the mechanism by which of N influences plant community structure has become an important area of research in community ecology (Niu et al., 2008; Hautier et al., 2009; Zhou et al., 2017). In particular, many studies have been done in an attempt to reveal how N addition changes natural plant community composition (Tilman, 1987; Dirkse and Martakis, 1992; Diekmann and Falkengren-Grerup, 2002). Some of them have focused on effects of N addition on interspecific competition for light and soil nutrients (Newman, 1973; Wilson and Tilman, 1993; Hautier et al., 2009) and others on how N addition influences plant functional traits and biomass allocation (Niu et al., 2008, 2009; Li et al., 2013). However, the link between germination traits and change in plant community composition due to N addition has not been explored.

Previous studies have shown that N addition has a significant effect on environmental conditions in the habitat and thus on community structure/composition (Tilman, 1987; Hobbs and Huenneke, 1992; Hautier et al., 2009). Nitrogen addition can increase nitrate (NO_3_^–^) and/or NH_4_^+^ in soil (Onipchenko et al., 2012), resulting in increased plant height and density (Tilman, 1987; Diekmann and Falkengren-Grerup, 2002). Increased plant cover can decrease daily maximum and minimum soil temperatures, maximum near-ground air temperature and magnitude of soil temperature fluctuations; however, it can increase the minimum near-ground air temperature and soil moisture (Zhao et al., 2011; Song et al., 2013). Furthermore, increased vegetation height and density can decrease solar irradiance and the red/far red ratio of light at the soil surface (Reed, 1977). All of these abiotic environmental factors can have a significant effect on seed germination (Steinbauer and Grigsby, 1957; Auchmoody, 1979; Freijsen et al., 1980; Sendon et al., 1986; Pons, 1989; Liu et al., 2013) and seeding establishment (Grime, 1973; Grime et al., 1981; Fenner and Thompson, 2005). Thus, we hypothesized that N addition can change the seed germination trait- environmental filter interactions and ultimately redistribute germination traits in alpine meadows, thereby causing changes in community composition.

To test our hypothesis, we tested for shifts in seed germination traits along a N addition gradient in Tibetan alpine meadows on the Tibet Plateau. At the species level after N addition, we expected (1) a broad temperature niche for germination, and (2) a positive germination response to temperature fluctuation. At the community level after N addition, we expected germination traits that would restrict germination to time and places (gaps) free from competition for light, would be more common in grasslands but that functional diversity of seed germination would decrease.

## Materials and Methods

### Study Site

The field experiment was conducted in an alpine meadow at the Research Station of Alpine Meadow and Wetland Ecosystems of Lanzhou University in Maqu (N33°40′, E101°52′, altitude 3,550 m), Gansu Province, China, on the eastern Qinghai-Tibet Plateau. Average annual precipitation (1983-2013) is 620 mm, and it occurs mainly during the short, cool summer (Zhang et al., 2014). Mean annual temperature 2°C, and mean monthly temperature varies from −10.7°C in January to 11.7°C in July. The annual cloud-free solar radiation is ∼2580 h (Luo et al., 2006). There is an average of 270 frost days per year. The alpine meadow is mainly dominated by *Anemone rivularis* (Ranunculaceae), *Carex kansuensis* (Cyperaceae), *Elymus nutans* (Poaceae), *Festuca ovina* (Poaceae), *Kobresia graminifolia* (Cyperaceae) and *Poa poophagorum* (Poaceae). All of these six species, except *P. poophagorum*, were included in our study.

### Nitrogen Addition Experiment and Vegetation Sampling

Five 10 m × 6 m blocks with the same topography and slope aspect separated by 2–3 m were established in a N-limited alpine meadow (Shen et al., 2002; Luo et al., 2006) in spring 2015. In each block, we established five 2 m × 4 m plots separated by 2 m and randomly allocated a control (N_0_, 0 g N m^–2^ year^–1^), low (N_1_, 5 g N m^–2^ year^–1^), intermediate (N_2_, 10 g N m^–2^ year^–1^) and high (N_3_, 20 g N m^–2^ year^–1^) plot. Thus, each of the four fertilization treatments included five replicates. The plots were fertilized on a day with light rainfall in early May 2015, 2016, and 2017 with granular slow-release ammonium nitrate (NH_4_NO_3_) applied by hand. Vegetation sampling was done at the peak of the growing season (July 2017). One 50 cm × 50 cm quadrat was sampled in each plot, and the presence and abundance (number of individuals of each species) of all species within this quadrat were recorded.

### Seed Collecting and Germination Experiments

Mature seeds of the 63 species found in the vegetation sample (Appendix S1) were collected at their natural dispersal time during July to October 2017. In general, seeds of each species were collected from more than 20 plants and from more than five non-fertilized grasslands located near the experimental site. If there were less than 20 plants of a species, we collected seeds from all plants of the species found. The collected seeds were used to test the three aspects of germination traits most related to plant survival in the field: (a) range of temperatures over which seeds would germinate, which can determine time during the growing season on the Tibet Plateau when seeds can germinate; (b) temperature fluctuation requirement for germination, which can restrict seed germination to vegetation gaps; and (c) cold stratification requirement for seed germination, which can determine whether seeds germinate before or after winter. There were two germination experiments. One tested the first two aspects of germination traits (experiment 1), and the other tested the third aspect of seed germination traits (experiment 2). Previous studies on the emergence of seedlings in the field have shown that most species in grasslands on the eastern Tibet Plateau have a long mean emergence time (MET, the period of time from seed dispersal to seedling emergence), indicating that seeds of most species in these communities need a period of after-ripening for germination (Cao et al., 2018). Therefore, seeds treated by after-ripening instead of fresh seeds were used in germination experiment 1. Thus, the seeds were air dried and cleaned, and stored under warm-dry conditions at room temperatures (ca. 15°C to 20°C), until used in experiments.

#### Experiment 1

Germination of seeds stored under dry-warm conditions was tested in incubators (Conviron E15 Growth Chamber, controlled Environments Ltd., Winnipeg, MB, Canada) at five constant temperatures (5, 10, 15, 20 and 25°C) and at an alternating temperature regime (5/25°C, 12/12h) in darkness. At the study site in the germination season (late March to late May in spring or late August to late October in autumn), the daily temperature range is about 5-25°C near the soil surface. Thus, the above constant temperatures and alternating temperature regime were selected in our study. This germination experiment was started in mid-March (when germination begins in the field) 2018, at which time seeds were 150 to 240 days old, depending on when they were collected, and any dormancy in fresh seeds would have bene broken via afterripening. For each species, there were three replicates of 50 seeds, and they were placed in Petri dishes (9-cm-diameter) on two sheets of filter paper moistened with distilled water. Germination was checked daily, at which time seeds were exposed to light for few minutes; thus, any light requirement for germination most likely was fulfilled during these exposures (Baskin and Baskin, 2014). Germinated seeds (radicle visible) were removed from the Petri dishes at each counting, and water was added to the filter paper as needed. The germination tests lasted for 60 days.

#### Experiment 2

To evaluate the effect of storage conditions on germination, we compared germination of seeds stored in dry-warm (at 15-20°C for 150-240 days, depending on when they were collected) and those receiving dry-warm + wet-cold (i.e., dry at room temperature for about 110-200 days depending on when they were collected and then on a moist substrate at 3°C for 40 days). For species requiring cold stratification to germinate, their main germinate season is spring (April to late May) (Cao et al., 2018), during which time the temperature ranges from 20°C in the day to 5°C at night (Liu et al., 2011). Thus, following the two storage treatments, seeds were incubated at 20°C (12 h)/5°C (12 h) in darkness for 60 days. The procedures for germination testing/monitoring were the same as those described above.

### Data Analysis

For the fertilization experiment, we calculated the response of species abundance to fertilization (R_*f*_). For the germination experiment at the species level, four germination indices were calculated: (1) germination percentage (GP); (2) germination rate (GR); (3) response of germination to alternating temperature (R_5__/__25_); and (4) germination response to wet-cold storage (R_*wc*_) (Table 1).

**TABLE 1:** Indices used in this study.

**Index**	**Meaning**	**Formula**	**Range**
R_*f*_	Response of species abundance to fertilization	R_*f*_ = (RAi_*N*__3_ - RAi_*N*__0_)/(RAi_*N*__3_ + RAi_*N*__0_) RAi_*N*__3_, RAi_*N*__0_: relative abundance of species i (i = 1, 2, 3 … 63) in N_3_ and N_0_, respectively.	[−1, 1]
RA_*i*_	Relative abundance of species	RA_*i*_ = n_*i*_/N n_*i*_: abundance of species i (i = 1, 2, 3 … 63); N: total number of all species.	[0, 1)
GP	Germination percentage	GP = (G**_*fin*_**_/_n) × 100% G**_*fin*_**: final number of germinated seeds; n: total number of seeds sown.	[0, 1]
GR	Germination rate	GR = ΣG**_*i*_/**(n × i) G**_*i*_**: number of germinated seeds on day i (i = 1, 2, 3…); n: total number of seeds sown.	[0, 1]
R_5__/__25_	Response of germination to alternating temperature	R_5__/__25_ = (GP_5__/__25_-GP_15_)/(GP_5__/__25_ + GP_15_) GP_5__/__25_, GP_15_: germination percentage at 5/25°C and 15°C, respectively.	[−1, 1]
R_*wc*_	Response of germination to wet-cold storage	R_*wc*_ = (GP_*wc*_ - GP_*dr*_)/(GP_*wc*_ + GP_*dr*_) GP_*wc*_, GP_*dr*_: germination percentage of seeds stored under wet-cold and dry-room conditions, respectively	[−1, 1]
BTN	Breadth of temperature niche for seed germination	BTN = 1/RΣO_*j*_ R: number of temperature conditions; O_*j*_: an index to measure the extent to which a given species occupies the *j*th temperature condition.	[1/R, 1] (R = 5)
O**_*j*_**	Species germination temperature occupation index	O**_*j*_** = g**_*j*_**/g**_*max*_** g**_*j*_**: germination percentage at *j*th temperature condition (j = 5, 10, 15, 20, 25); g**_*max*_**: highest germination percentage at all temperature conditions.	[0, 1]
P**_*j*_**	Proportion of germination temperature niche	P**_*j*_** = O**_*j*_**/ΣO**_*j*_** O**_*j*_**: species germination temperature occupancy index.	[0, 1]
CWM_*trait*_ **_*i*_**	Community-weighted means of germination traits index	CWM_*trait*_ **_*i*_** = Σtrait**_*i*_** × RA**_*i*_** trait **_*i*__:_** germination trait index (trait = GPi, GRi, R_5__/__25_, R_*wc*_, P**_*j*_**, O**_*j*_**, BTN); RA**_*i*_**: relative abundance of species i (i = 1, 2, 3 … 63).	(0, ∞)

Three indices (Table 1) were calculated to describe the seed germination niche: BTN, breadth of temperature niche for seed germination; O_*j*_, the extent to which a given species occupies the *j*th germination temperature condition (j = 5, 10, 15, 20, 25); P_*j*_, proportion of temperature niche for seed germination at the *j*th temperature condition (j = 5, 10, 15, 20, 25).

To compare seed germination traits at the community level, community-weighted means (CWM) of these indices of germination traits were calculated by weighting these indices with species relative abundance to avoid amplifying the influence of rare species in the community (Niu et al., 2016a, c; Table 1). Germination trait diversity (the range and variation of germination indices in a community) was tested based on seven variables (seed germination percentage at 5, 10, 15, 20 and 25°C and response of seed germination to temperature fluctuation and wet-cold storage) using the ‘dbFD’ function of the R package ‘FD’ (Laliberté and Legendre, 2010). Three typical diversity indices (Mason et al., 2005; Villéger et al., 2008; Niu et al., 2016b) were estimated: 1) FRic, functional richness; 2) FEve, functional evenness; and 3) FDiv, functional divergence.

Beta regression, a model for response variable bounded in (0,1), was used to test the correlation of GP, GR, R_5__/__25_, R_*wc*_, BTN, O_*j*_ and P_*j*_ with R_*f*_ (Ferrari and Cribari-Neto, 2004). Simultaneously, to meet the requirements of Beta regression we removed the boundary values (i.e., −1 and 1) in the data and converted the data with (y + 1)/2 to shift our data interval from (−1, 1) to (0, 1) (Douma and Weedon, 2019). The Beta regression was performed with the ‘betareg’ function of the R package ‘betareg’ (Cribari-Neto and Zeileis, 2010). Generalized linear mixed model (GLMM) was used to evaluate the effects of N addition, temperature and their interaction on community-weighted mean of each germination trait (i.e., CWM_*GP*_, CWM_*GR*_, CWM_*R*__5__/__25_, CWM_*Rwc*_, CWM_*BTN*_, CWM_*Oj*_ and CMN_*Pj*_) and germination trait diversity indices (FRic, FEve and FDiv), because of the lack of normality and homogeneity of the data. When GLMM was conducted, block was treated as a random variable. GLMM was conducted with the ‘glmer’ function of the R package ‘lme4’ (Bates et al., 2015) on communities with different N additions at different temperatures. Comparison of data between groups was performed by Tukey test.

To determine if shifts in seed germination traits occurred along the fertilization gradient, we used Principal Components Analysis (PCA), based on a correlation matrix, to identify species groupings or trends on the basis of comprehensive variables. CWM of 23 variables were included in PCA: CWM_*GP*_ at 5, 10, 15, 20 and 25°C; CWM_*GR*_ at 5, 10, 20 and 25°C; CWM of breadth of temperature niche for germination; CWM of occupation of temperature niche for germination at 5, 10, 15, 20 and 25°C; CWM of proportion of temperature niche for seed germination at 5, 10, 15, 20 and 25°C; CWM of R_5__/__25_; and CWM of R_*wc*_. PCA was conducted using the ‘princomp’ function of the R package ‘stats’ (R Core Team, 2021). All statistical analyses were conducted using R version 4.0.4.

## Results

### Relationship Between Germination (GP and GR) and Response of Species Abundance to Nitrogen Fertilization (R_*f*_)

Relative abundance of species along the fertilization gradient is shown in Appendix S2. The results of Beta regression analysis indicated that germination percentages at the five temperatures (5, 10, 15, 20 and 25°C) were significantly and positively related to R_*f*_. Germination rates of seeds at 10, 15, 20 and 25°C were significantly related to R_*f*_ (Table 2), whereas at 5°C they were not (Table 2).

**TABLE 2:** Results of Beta regression models testing correlations of response of species abundance to fertilization (R_*f*_) with germination percentage, germination rate (speed) and response of germination percentage to temperature fluctuation and wet-cold storage.

**Germination index**	**Regression equation**	**z-value**	***P*-value**	***R*^2^**
GP_5_	y = −0.96 + 1.44x	3.286	**0.001**	0.263
GP_10_	y = −0.83 + 1.51x	3.382	**< 0.001**	0.271
GP_15_	y = −0.84 + 1.34x	3.037	**0.002**	0.224
GP_20_	y = −1.05 + 1.41x	3.256	**0.001**	0.256
GP_25_	y = −1.03 + 1.34x	2.986	**0.003**	0.218
GP_5_/_25_	y = −0.64 + 0.79x	1.781	0.075	0.092
GR_5_	y = −0.68 + 19.81x	2.323	0.20	0.151
GR_10_	y = −0.78 + 13.31x	3.505	**< 0.001**	0.292
GR_15_	y = −0.71 + 7.03x	2.949	**0.003**	0.230
GR_20_	y = −0.69 + 3.67x	2.678	**0.007**	0.180
GR_25_	y = −0.83 + 4.81x	3.160	**0.002**	0.257
GR_5__/__25_	y = −0.63 + 7.05x	2.182	**0.029**	0.129
R_5/25_	y = −0.28 + (-0.23)x	−0.858	0.391	0.023
R_*wc*_	y = −0.29 + (-0.19)x	−0.573	0.567	0.011

*Significant *P* values (*P* < 0.05) are indicated in bold. GP_5_, GP_10_, GP_15_, GP_20_, GP_25_ and GP_5/25_: germination percentage at 5, 10, 15, 20, 25 and 5/25 °C, respectively; GR_5_, GR_10_, GR_15_, GR_20_, GR_25_ and GR_5/25_: germination rate at 5, 10, 15, 20, 25 and 5/25°C, respectively. R_5/25_: response of germination to temperature fluctuation; R_*wc*_: response of germination to wet-cold storage.*

**FIGURE 1:**
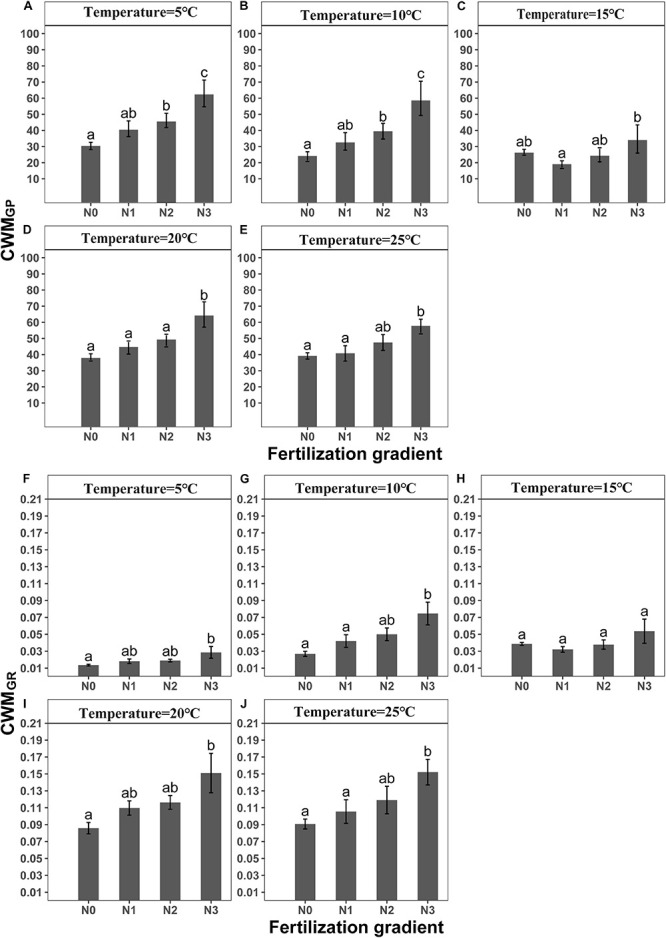
Effects of temperature and level of nitrogen addition (N0, 0 g N m ^–2^ year ^–1^; N1, 5 g N m^–2^ year^–1^; N2, 10g N m^–2^ year^–1^; N3, 20g N m^–2^ year^–1^) on CWM_*GP*_ (community weighted mean of germination percentage) and **(A–E)** CWM_*GR*_ (community weighted mean of germination rate). **(F–J)** Different letters indicate significant differences in level of nitrogen addition (*P* < 0.05) at each temperature. Error bars are ± SE.

GLMM indicated that both temperature (5, 10, 15, 20 and 25°C) and fertilization (N_0_, N_1_, N_2_ and N_3_) had a significant effect on community-weighted means for germination percentages and rates (CWM_*GP*_ and CWM_*GR*_) (Table 3). There were no interaction effects between temperature and fertilization on CWM_*GP*_ or CWM_*Gr*_ (Table 3). CWM_*GP*_ increased with increased N addition, and CWM_*GP*_ was significantly higher in the N_3_ treatment than in the N_0_ treatment (Figure 1). Only under low temperature (5 and 10°C), CWM_*GP*_ was significantly higher in the N_2_ treatment than in the N_0_ treatment (Figure 1). Similarly, CWM_*G*__*R*_ increased along the fertilization gradient (except at 15°C), where there was a significant difference between N_3_ and N_0_ (Figure 1).

**TABLE 3:** Results Generalized linear mixed model (GLMM) testing the effect of temperature (tem), fertilization gradient (fer) and their interaction on community weighted mean of germination percentage (CWM_*GP*_) and of germination rate (CWM_*GR*_).

**Response/AIC/BIC**	**Fixed effects**		
	**χ 2**	**DF**	***P*-value**
CWM_*GP*_/673.4/728.1	139.76	4	**<0.001**
Temperature	161.49	3	**<0.001**
Fertilization	25.12	12	**0.014**
temperature*fertilization			
CWM_*GR*_/351.1/405.8			
Temperature	105.50	4	**<0.001**
Fertilization	16.48	3	**<0.001**
temperature*fertilization	1.97	12	0.999

*Significant *P* values (*P* < 0.05) are indicated in bold.*

### Relationship Between Germination Response to Alternating Temperature (R_5__/__25_), Cold-wet Storage (R_*wc*_) and R_*f*_

**FIGURE 2:**
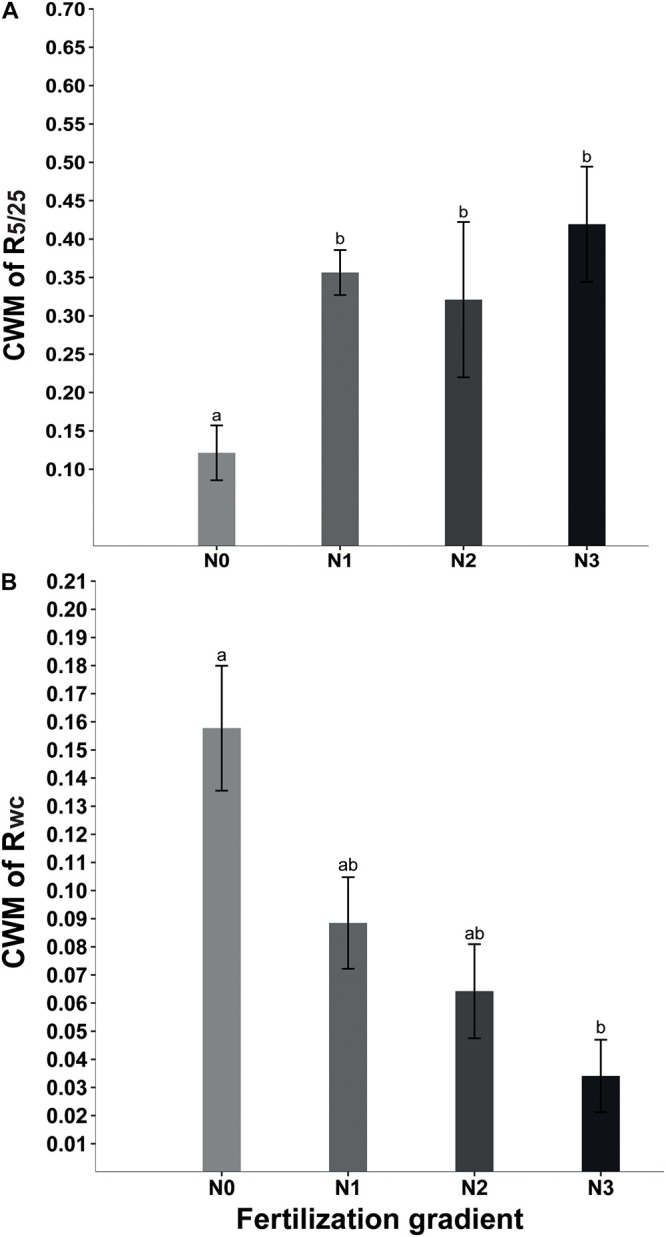
Effect of nitrogen addition (N0, 0 g N m^–2^ year^–1^; N1, 5 g N m^–2^ year^–1^; N2, 10g N m^–2^ year^–1^; N3, 20g N m^–2^ year^–1^) on CWM (community weighted mean) of R_5/25_ (germination response to temperature fluctuation) **(A)** and CWM of R_*wc*_ (germination response to wet-cold storage) **(B)**. Different letters indicate significant differences (*P* < 0.05). Error bars are ± SE.

Beta regression analysis showed no significant relationship between R_5__/__25_ and R_*f*_ (Table 2). Neither did R_*wc*_ have a significant relationship with R_*f*_ (Table 2). However, at the community level GLMM showed that fertilization significantly affected CWM_*R*__5__/__25_ (*x*^2^ = 54.39, *P* < 0.001) and CWM_*Rwc*_ (*x*^2^ = 15.29, *P* = 0.002). The strength of the positive response of seed germination to alternating temperature was significantly higher in N_1_, N_2_, and N_3_ treatments than in N_0_ treatment (Figure 2A). In contrast, at the community level the strength of the positive response of seed germination to wet-cold storage decreased with increase in amount of N added, and CWM_*wc*_ was significantly lower in N_3_ treatments than in N_0_ treatment (Figure 2B).

### Relationship Between Temperature Niche for Seed Germination (BTN) and R_*f*_

**FIGURE 3:**
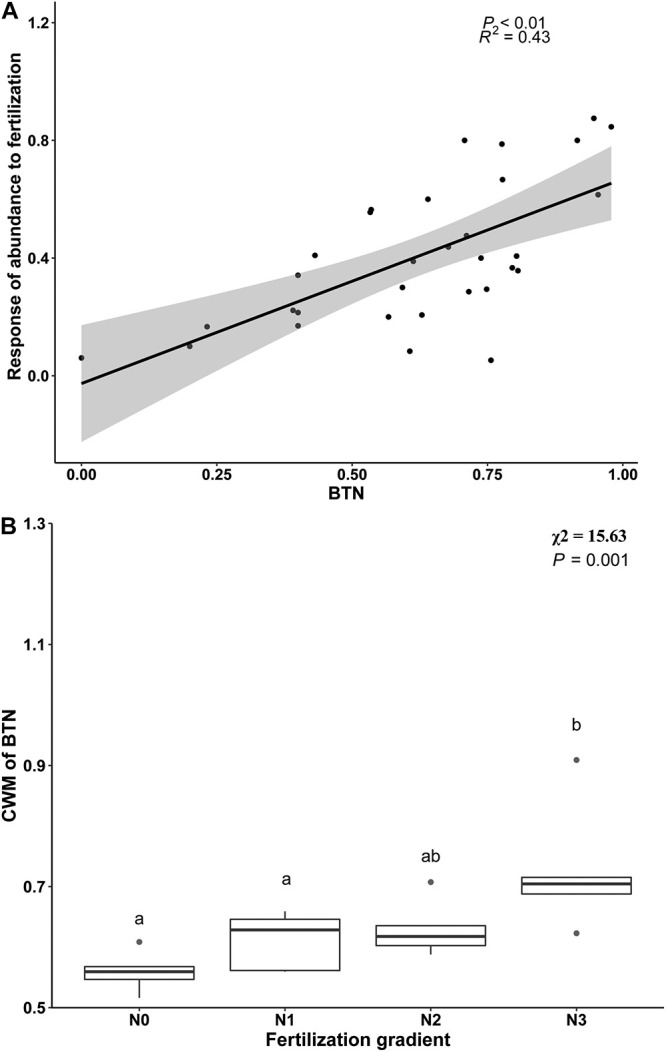
The relationship between response of species abundance to fertilization (R_*f*_) and breadth of temperature niche for germination (BTN) at species level **(A)** and the effect of nitrogen addition (N0, 0 g N m^–2^ year^–1^; N1, 5 g N m^–2^ year^–1^; N2, 10g N m^–2^ year^–1^; N3, 20g N m^–2^ year^–1^) on CWM (community weighted mean) of BTN **(B)**. Ends of a box represent first and third quartiles; thick line in box, the median; ends of vertical line (whiskers), maximum and minimum; and dots, outliers.

Beta regression analysis revealed a significant positive relationship between BTN and R_*f*_ (Figure 3A), and it also showed that proportion of temperature niche for seed germination at 10°C (P_10_) was significantly and positively related to R_*f*_ (Table 4). However, there was no significantly positive relationship between R_*f*_ and proportion of temperature niche for seed germination at the other temperatures (Table 4). The results of Beta regression analysis indicated that occupation index of seed germination for all temperature conditions (5, 10, 15, 20 and 25°C) was always significantly and positively related to R_*f*_ (Table 4). At the community level, the CWM_*BTN*_ of the community treated with N_3_ was significantly higher than that of the control (Figure 3B). The CWM_*BTN*_ of the community with N_1_ or N_2_ did not differ significantly from N_0_ or N_3_ (Figure 3B).

**TABLE 4:** Results of Beta regression models testing correlations of response of species abundance to fertilization (R_*f*_) with traits of temperature niche for germination.

**Niche traits**	**Regression equation**	***z*-value**	***P*-value**	***R*^2^**
O_*i*__5_	y = −1.06 + 1.10x	2.530	**0.011**	0.202
O_*i*__10_	y = −1.12 + 1.68x	4.038	**<0.001**	0.348
O_*i*__15_	y = 0.81 + 0.85x	1.988	**0.047**	0.111
O_*i*__20_	y = −1.13 + 1.02x	1.971	**0.049**	0.138
O_*i*__25_	y = −1.15 + 1.02x	2.111	**0.035**	0.143
P_*i*__5_	y = −0.63 + 1.45x	1.130	0.258	0.047
P_*i*__10_	y = −1.02 + 5.51x	3.084	**0.002**	0.244
P_*i*__15_	y = −0.49 + 0.92x	0.622	0.534	0.013
P_*i*__20_	y = −0.29 + (-0.26)x	−0.252	0.801	0.003
P_*i*__25_	y = −0.24 + (-0.43)x	−0.464	0.642	0.010

*Significant *P* values (*P* < 0.05) are indicated in bold. BTN: breadth of temperature niche for germination; O_5_, O_10_, O_15_, O_20_ and O_25_: occupation of temperature niche for germination at 5, 10, 15, 20 and 25°C, respectively; P_5_, P_10_, P_15_, P_20_ and P_25_: proportion of temperature niche for seed germination at 5, 10, 15, 20 and 25°C, respectively.*

### Principal Component Analysis (PCA) of Seed Germination Strategies at Different Nitrogen Levels

**FIGURE 4:**
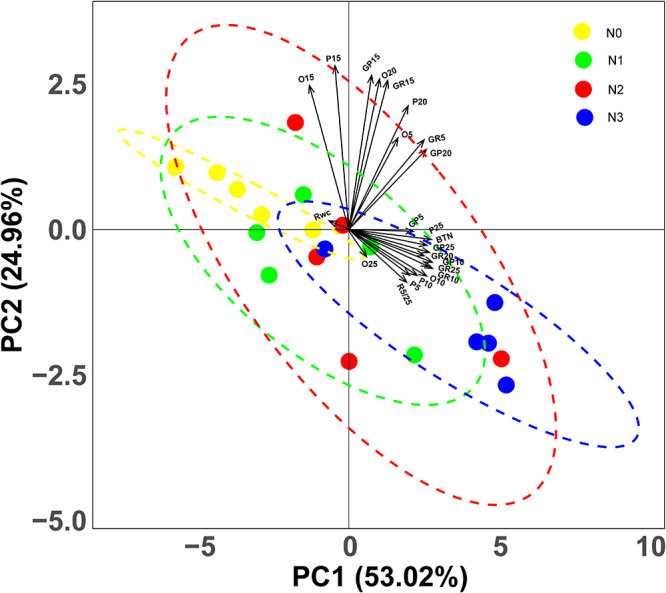
Principal Component Analysis (PCA) representing the two main axes of variation in germination traits, based on community weighted mean (CWM) of 23 variates. GP5, GP10, GP15, GP20 and GP25: germination percentage at 5, 10, 15, 20 and 25°C respectively; GR5, GR10, GR20 and GR25: germination rate at 5, 10, 15, 20 and 25°C, respectively; BTN: breadth of temperature niche for germination; O5, O10, O15, O20 and O25: occupation of temperature niche for germination at 5, 10, 15, 20 and 25°C, respectively; P5, P10, P15, P20 and P25: proportion of temperature niche for seed germination 5, 10, 15, 20 and 25°C, respectively. Dots represent quadrats, different colors represent different levels of nitrogen addition.

Based on the CWM of 23 variables, PCA revealed some clear trends. The first two principal components (PCs) accounted for 77.98% of the variation in the data set, with the first and second PCs explaining 53.02 and 24.96% of the variation, respectively. The two PCs represent the linear combinations of variables that are highly correlated with each other (Figure 4). The three variables most positively related to the first PC are CWM_*GP*__5_, CWM_*P*__25_ and CWM_*BTN*_; however, the variable most negatively related to the first PC is CWM_*RWC*_. The three variables most positively related to the second PC are CWM_*O*__15_, CWM_*Gp*__15_ and CWM_*P*__15_, while the three variables most negatively related to the second PC are CWM_*P*__5_, CWM_*R*__5__/__25_ and CWM_*O*__25_ (Figure 4). PCA revealed that shifts of germination strategies occurred along the entire fertilization gradient and especially in the high N addition treatment (N_3_) (Figure 4).

### Shift in Diversity of Germination Traits Along the Nitrogen Fertilization Gradient

**FIGURE 5:**
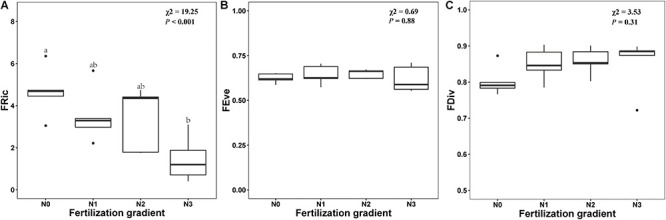
Effects of level of nitrogen addition (N0, 0 g N m^–2^ year^–1^; N1, 5 g N m^–2^ year^–1^; N2, 10g N m^–2^ year^–1^; N3, 20g N m^–2^ year^–1^) on functional richness (FRic, **A)**, functional evenness (FEve, **B)** and functional divergence (FDiv, **C)** of germination. Ends of a box represent first and third quartiles; thick line in box, the median; ends of vertical line (whiskers), maximum and minimum; and dots, outliers.

Germination trait diversity indices along the N fertilization gradient are shown in Appendix S3. GLMM indicated that N addition had a significant effect on FRic (*x*^2^ = 19.25, *P* < 0.001). FRic decreased with increased N, and FRic was significantly lower in N_3_ treatments than in N_0_ treatment (Figure 5A). However, N addition did not have a significant effect on either FEve or FDiv (*P* = 0.88 and *P* = 0.31, respectively) (Figures 5B,C).

## Discussion

### Changes in Germination Trait Selection Due to Fertilization

Our hypothesis that changes occurring after N addition can change seed germination trait-environmental filter interactions and ultimately redistribute germination traits in the community and alter community composition was supported. At the species level, germination percentage at all temperatures, germination rate at all test temperatures except 5 °C and properties of the temperature niche for germination (BTN, P_5_, O_*j*_) were significantly related to R_*f*_. Analyses at the community level also indicated significant differences between CWM_*GP*_, CWM_*GR*_, CWM_*R*__5__/__25_, CWM_*Rwc*_ and CWM_*BTN*_ of the alpine meadow with high level of N addition and those without N addition. The seed germination trait richness of the alpine meadow also decreased significantly after N addition. These effects of N addition on germination trait selection by the environmental filter is amount-dependent (i.e., low and medium level of N addition had less effect than the high level).

These results indicated that N addition alters filter-trait interactions. The environmental filter in the alpine meadows after N addition tended to select germination traits such as high germination percentage, fast germination rate (speed) and an alternating temperature requirement for germination. However, other germination traits were not selected by the environment filter (N addition), resulting in a decrease of seed germination trait richness in the meadow.

Previous studies indicated that fertilization can cause loss of biodiversity in the grassland community (Niu et al., 2008; Hautier et al., 2009). The decrease in germination traits richness in our study may provide a new explanation for biodiversity loss in grasslands. That is, the environmental filter (N fertilization) does not select for as many germination traits as found in grasslands without fertilization. Thus, grassland species with germination traits not selected by the fertilization filter may be lost from grasslands after fertilization.

### Germination Traits and Plant Survival in Alpine Meadows After Nitrogen Addition

Our research provides some evidence that seed germination traits at both the species and community levels play a role in plant survival/persistence in alpine meadows after N addition. Species with high germination percentages and rates are more likely to survive in fertilized alpine meadows than those with low germination percentages and rates. Previous studies indicated that germination traits such as high germination percentage and fast germination speed tend to be selected by a favorable environment (Baskin and Baskin, 2014). The increased plant height and density after N addition can increase soil moisture and reduce exposure to strong sunlight on the soil surface (Bakelaar and Odum, 1978; Ostertag and Verville, 2002; Fenner and Thompson, 2005; Song et al., 2013). In addition, N addition increases soil nutrition (Niu et al., 2008; Li et al., 2013). These habitat changes can promote seedling growth and survival and decrease the possibility of seedling death due to high temperature and drought conditions (Fenner and Thompson, 2005). Thus, favorable germination traits for species to occupy space and acquire resources, such as high germination percentage and fast germination speed, may be selected in fertilized alpine meadows.

Competition for light is a primary determinant of the survival of plant species in the community after N addition (Grime, 1973; Newman, 1973; Tilman, 1988). Since competition for light is plant size- and height-dependent, a newly-germinated seedling in a high-N community is at a great disadvantage with established plants in capturing light. Seed germination traits that can restrict germination to a time and place without competition for light are more likely be selected by the fertilization environmental filter in alpine meadows. Our study showed that the magnitude (slope) of the relationship between seed germination rate and response of species relative abundance to N addition is much greater at low than that at high temperatures. Thus, faster germination speed at low than at high temperature is important for survival of plant species in fertilized alpine meadows. Fast germination in early spring (when the temperature is low on the Tibet Plateau) is conducive to successful seedling establishment before the canopy of established plants becomes dense in the spring. It is a mechanism by which a plant species can avoid seedling death due to competition for light. Furthermore, the magnitude (slope) of the relationship between occupation of temperature niche for germination at low temperature (10°C) and the response of abundance to N fertilization (R_*f*_) is larger than that at high temperature, and a proportion of germination temperature niche can positively relate to the response of abundance to fertilization (R_*f*_), only at low temperature (10°C). These two results also prove that seed germinability at low temperatures is critical for plant species survival in fertilized alpine meadows. Previous studies also found that herbs in dense vegetation usually can germinate in early spring at low temperatures (Baskin and Baskin, 1988; Schütz, 1997; Vandelook et al., 2009).

As mentioned above, competition for light after formation of a dense canopy in spring is not conducive to seedling establishment. However, in Tibetan alpine meadows many vegetation gaps usually are created by burrowing animals such as pika, zokor and marmot and by overgrazing by sheep and yak (Liu et al., 2011). Light is sufficient for seedling establishment in these vegetation gaps. A fluctuating temperature requirement for germination, which restricts germination to vegetation gaps, would promote survival of plant species in fertilized alpine meadows. Our study indicates that at the community level, N addition significantly increased germination response to alternating temperature; seeds of more species in the fertilized than in the non-fertilized meadow required alternating temperatures to germinate. Since addition of N promotes plant growth and canopy closure, we would expect gap-sensing mechanisms for successful seedling establishment to be more important in fertilized than in non-fertilized alpine meadows. As expected, there was a stronger positive response of seed germination to alternating temperatures of species in fertilized than in non-fertilized alpine meadows.

A broad BTN is another germination trait that can promote survival of plant species in fertilized alpine meadows. In our research, Beta regression analysis revealed a significant positive relationship between BTN and R_*f*_. One reason for this positive relationship is that a broad BTN allows seeds to germinate as soon as a vegetation gap is created, and a fluctuating temperature requirement for germination is fulfilled during the growing season, which results in an advantage for seedlings to occupy vegetation gaps, where the light environment is suitable for growth and establishment. Another reason is that seeds of species with a broad temperature niche for germination usually can germinate to high percentages at 5°C and 10°C, which may be beneficial for seedling establishment before the canopy becomes too dense in early spring.

## Conclusion

Our study indicates that N addition has a significant influence on filter-trait interactions and can generate a different set of germination traits in alpine meadows. As a result, germination trait richness was decreased by N addition to the alpine meadow. In addition, the effect of N addition on germination trait selection by the environmental filter was amount-dependent (i.e., low and medium levels of N addition had less effect than the high level). A broad temperature niche, a requirement of alternating temperatures for germination, a high germination percentage and a fast germination rate, especially at low temperature, are germination traits more likely to be selected for in fertilized than in non-fertilized alpine meadows. Additional studies on the effect of N addition on seedling establishment and plant clonal reproduction at the species and community levels will help us to further evaluate the extent to which germination traits are responsible for plant survival in alpine meadows that have become N-enriched via use of N fertilizers by humans or via atmospheric N deposition.

## Data Availability Statement

The original contributions presented in the study are included in the article/Supplementary Material, further inquiries can be directed to the corresponding author/s.

## Author Contributions

KL, YL, ZZ, and SZ started the project, designed the research, KL, YL, CB, and JB made major revision of the manuscript. KL, YL, ZZ, SZ, HB, TL, SL, and TZ performed the research. KL, YL, XC, and SX analyzed data and drafted the manuscript. All authors contributed to the article and approved the submitted version.

## Conflict of Interest

The authors declare that the research was conducted in the absence of any commercial or financial relationships that could be construed as a potential conflict of interest.

## Supplementary Material

Supplementary Appendix 1 Germination percentages of seeds of the 63 species tested in the study. GP_5_, GP_10_, GP_15_, GP_20_, GP_25_ and GP_(5/__25__)_: germination percentage at 5°C, 10°C, 15°C, 20°C, 25°C and 5/25°C, respectively; GP_*dr*_ and GP_*wc*_: germination percentage at 5/20°C of seeds stored under day-warm condition and wet-cold conditions, respectively.

Supplementary Appendix 2 Relative abundance of species along the nitrogen fertilization gradient. RaN0, RaN1, RaN2 and RaN3: relative abundance of species (Ra) at positions along the gradient where soil received an application of 0 (N0), 5 (N1), 10 (N2) and 20 (N3) g N m^–2^y^–1^.

Supplementary Appendix 3 Germination trait diversity indices (functional richness, FRic; functional evenness, FEve; and functional divergence, FDiv) at different levels of nitrogen addition along the nitrogen fertilization gradient.
