# Supplementary material for: Effect of Nitrogen Addition on Selection of Germination Trait in an Alpine Meadow on the Tibet Plateau
Source: Front Plant Sci. 2021 May 14;12:634850. doi: 10.3389/fpls.2021.634850 (PMC8160428; doi:10.3389/fpls.2021.634850)
Supplement: Supplementary Appendix 1 — Germination percentages of seeds of the 63 species tested in the study. GP5, GP10, GP15, GP20, GP25 and GP(5/25): germination percentage at 5°C, 10°C, 15°C, 20°C, 25°C and 5/25°C, respectively; GPdr and GPwc: germination percentage at 5/20°C of seeds stored under day-warm condition and wet-cold conditions, respectively. [file Data_Sheet_1.doc]

**Appendix S1. Germination percentages of seeds of the 63 species tested in the study. GP_5_, GP_10_, GP_15_, GP_20_, GP_25_ and GP_(5/25)_: germination percentage at 5℃, 10℃, 15℃, 20℃, 25℃ and 5/25°C, respectively; GPdr and GP_wc_: germination percentage (at 5/20°C) of seeds stored under day-warm condition and wet-cold conditions, respectively.**

| **Species** | **Family** | **GP5** | **GP10** | **GP15** | **GP20** | **GP25** | **GP5/25** | **GPdr** | **GPwc** |
| --- | --- | --- | --- | --- | --- | --- | --- | --- | --- |
| *Adenophora himalayana* | Campanulaceae | 0.01 | 0.37 | 0.49 | 0.63 | 0.69 | 0.95 | 0.45 | 0.20 |
| *Agrostis hugoniana* | Poaceae | 0.13 | 0.05 | 0.65 | 0.49 | 0.71 | 0.91 | 0.87 | 0.53 |
| *Agrostis trinii* | Poaceae | 0.47 | 0.02 | 0.74 | 0.73 | 0.73 | 0.24 | 0.77 | 0.63 |
| *Ajuga lupulina* | Lamiaceae | 0.53 | 0.00 | 0.00 | 0.00 | 0.00 | 0.00 | 0.05 | 0.01 |
| *Allium sikkimense* | Amaryllidaceae | 0.27 | 0.29 | 0.39 | 0.93 | 0.89 | 0.82 | 0.85 | 1.00 |
| *Anemone obtusiloba* | Ranunculaceae | 0.01 | 0.00 | 0.00 | 0.17 | 0.02 | 0.11 | 0.03 | 0.57 |
| *Anemone rivularis* | Ranunculaceae | 0.74 | 0.00 | 0.01 | 1.00 | 0.41 | 0.14 | 0.15 | 0.98 |
| *Anemone trullifolia* | Ranunculaceae | 0.00 | 0.00 | 0.01 | 0.01 | 0.00 | 0.00 | 0.00 | 0.03 |
| *Angelica nitida* | Apiaceae | 0.04 | 0.01 | 0.01 | 0.17 | 0.03 | 0.01 | 0.00 | 0.53 |
| *Artemisia tangutica* | Asteraceae | 0.30 | 0.05 | 0.62 | 0.87 | 0.81 | 0.50 | 0.73 | 0.67 |
| *Artemisia mongolica* | Asteraceae | 0.51 | 0.27 | 0.52 | 0.52 | 0.51 | 0.23 | 0.80 | 0.85 |
| *Aster diplostephioides* | Asteraceae | 0.91 | 0.81 | 0.80 | 0.82 | 0.73 | 0.47 | 0.92 | 0.83 |
| *Aster souliei* | Asteraceae | 0.19 | 0.04 | 0.07 | 0.11 | 0.25 | 0.05 | 0.05 | 0.61 |
| *Astragalus ploycladus* | Fabaceae | 0.21 | 0.11 | 0.16 | 0.26 | 0.24 | 0.65 | 0.21 | 0.08 |
| *Carex crebra* | Cyperaceae | 0.00 | 0.00 | 0.00 | 0.00 | 0.42 | 0.05 | 0.00 | 0.02 |
| *Carum buriaticum* | Apiaceae | 0.45 | 0.14 | 0.78 | 0.83 | 0.87 | 0.17 | 0.46 | 1.00 |
| *Cerastium fontanum* | Caryophyllaceae | 0.77 | 0.62 | 0.59 | 0.61 | 0.85 | 0.17 | 0.36 | 0.87 |
| *Delphinium kamaonense* | Ranunculaceae | 0.65 | 0.53 | 0.75 | 0.61 | 0.95 | 0.83 | 0.99 | 0.97 |
| *Elymus nutans* | Poaceae | 0.91 | 0.88 | 0.03 | 0.84 | 0.89 | 0.91 | 0.94 | 0.97 |
| *Euphrasia regelii* | Orobanchaceae | 0.00 | 0.00 | 0.01 | 0.00 | 0.00 | 0.00 | 0.01 | 0.43 |
| *Festuca ovina* | Poaceae | 0.68 | 0.66 | 0.82 | 0.80 | 0.83 | 0.60 | 0.94 | 0.95 |
| *Galium verum* | Rubiaceae | 0.21 | 0.16 | 0.31 | 0.62 | 0.60 | 0.94 | 0.90 | 0.89 |
| *Gentiana aristata* | Gentianaceae | 0.00 | 0.03 | 0.00 | 0.00 | 0.00 | 0.02 | 0.00 | 0.10 |
| *Gentiana farreri* | Gentianaceae | 0.61 | 0.00 | 0.54 | 0.65 | 0.52 | 1.00 | 0.61 | 0.69 |
| *Gentiana straminea* | Gentianaceae | 0.83 | 0.19 | 0.84 | 0.52 | 0.93 | 0.35 | 0.07 | 0.68 |
| *Gentianopsis paludosa* | Gentianaceae | 0.00 | 0.00 | 0.01 | 0.03 | 0.00 | 0.00 | 0.03 | 0.04 |
| *Geranium pylzowianum* | Geraniaceae | 0.33 | 0.09 | 0.22 | 0.18 | 0.31 | 0.03 | 0.73 | 0.04 |
| *Gueldenstaedtia verna* | Fabaceae | 0.03 | 0.09 | 0.11 | 0.12 | 0.11 | 0.17 | 0.41 | 0.07 |
| *Ixeris polycephala* | Asteraceae | 0.80 | 0.65 | 0.01 | 0.64 | 0.46 | 0.85 | 0.89 | 0.79 |
| *Kobresia graminifolia* | Cyperaceae | 0.04 | 0.01 | 0.01 | 0.01 | 0.01 | 0.05 | 0.11 | 0.08 |
| *Kobresia tibetica* | Cyperaceae | 0.00 | 0.00 | 0.03 | 0.01 | 0.04 | 0.23 | 0.07 | 1.00 |
| *Koeleria litvinowii* | Poaceae | 0.25 | 0.32 | 0.52 | 0.82 | 0.58 | 0.40 | 0.17 | 0.95 |
| *Leontopodium calocephalum* | Asteraceae | 0.95 | 0.98 | 0.99 | 0.97 | 0.89 | 0.97 | 0.93 | 0.91 |
| *Leontopodium souliei* | Asteraceae | 0.87 | 0.98 | 0.93 | 0.88 | 0.89 | 0.86 | 0.83 | 0.91 |
| *Ligularia virgaurea* | Asteraceae | 0.82 | 0.83 | 0.59 | 0.71 | 0.00 | 0.80 | 0.76 | 0.59 |
| *Lomatogonium carinthiacum* | Gentianaceae | 0.00 | 0.00 | 0.01 | 0.01 | 0.03 | 0.57 | 0.00 | 0.97 |
| *Morina chinensis* | Dipsacaceae | 0.00 | 0.03 | 0.00 | 0.15 | 0.00 | 0.02 | 0.05 | 0.91 |
| *Oxytropis kansuensis* | Fabaceae | 0.02 | 0.04 | 0.08 | 0.27 | 0.43 | 0.04 | 0.13 | 0.07 |
| *Parnassia trinervis* | Celastraceae | 0.53 | 0.27 | 0.47 | 0.67 | 0.57 | 0.85 | 0.67 | 0.97 |
| *Pedicularis cranolopha* | Orobanchaceae | 0.59 | 0.27 | 0.27 | 0.27 | 0.29 | 0.22 | 0.10 | 0.78 |
| *Pedicularis kansuensis* | Orobanchaceae | 0.30 | 0.28 | 0.69 | 0.79 | 0.90 | 0.52 | 0.85 | 0.15 |
| *Plantago asiatica* | Plantaginaceae | 0.85 | 0.44 | 0.13 | 0.27 | 0.73 | 0.33 | 0.59 | 0.93 |
| *Plantago depressa* | Plantaginaceae | 0.81 | 0.01 | 0.77 | 0.55 | 0.88 | 0.65 | 0.33 | 0.97 |
| *Pleurospermum hookeri* | Apiaceae | 0.00 | 0.00 | 0.00 | 0.00 | 0.00 | 0.08 | 0.01 | 1.00 |
| *Poa crymophila* | Poaceae | 0.77 | 0.81 | 0.78 | 0.80 | 0.85 | 0.95 | 0.55 | 0.76 |
| *Poa pachyantha* | Poaceae | 0.79 | 0.80 | 0.97 | 0.92 | 0.95 | 0.90 | 0.98 | 0.82 |
| *Potentilla anserina* | Rosaceae | 0.00 | 0.00 | 0.00 | 0.00 | 0.00 | 0.00 | 0.03 | 0.08 |
| *Potentilla saundersiana* | Rosaceae | 0.21 | 0.09 | 0.56 | 0.63 | 0.69 | 0.22 | 0.40 | 0.27 |
| *Ranunculus tanguticus* | Ranunculaceae | 0.02 | 0.00 | 0.00 | 0.00 | 0.02 | 0.21 | 0.47 | 0.16 |
| *Rumex acetosa* | Polygonaceae | 0.29 | 0.22 | 0.37 | 0.27 | 0.30 | 0.16 | 0.75 | 0.54 |
| *Saussurea stella* | Asteraceae | 0.99 | 0.93 | 0.97 | 0.95 | 0.90 | 1.00 | 0.95 | 0.98 |
| *Saussurea hieracioides* | Asteraceae | 0.85 | 0.81 | 0.85 | 0.79 | 0.63 | 0.80 | 0.98 | 0.77 |
| *Saussurea nigrescens* | Asteraceae | 0.59 | 0.66 | 0.57 | 0.53 | 0.79 | 0.69 | 0.89 | 0.91 |
| *Saussurea pachyneura* | Asteraceae | 0.66 | 0.60 | 0.57 | 0.56 | 0.63 | 0.50 | 0.25 | 0.79 |
| *Scirpus distigmaticus* | Cyperaceae | 0.00 | 0.00 | 0.00 | 0.00 | 0.00 | 0.00 | 0.00 | 0.00 |
| *Scutellaria baicalensis* | Lamiaceae | 0.24 | 0.03 | 0.25 | 0.24 | 0.07 | 0.42 | 0.61 | 0.34 |
| *Stellaria uda* | Caryophyllaceae | 0.91 | 1.00 | 0.99 | 1.00 | 0.99 | 0.30 | 0.97 | 0.97 |
| *Stipa aliena* | Poaceae | 0.97 | 0.68 | 0.66 | 0.64 | 0.68 | 0.16 | 0.89 | 1.00 |
| *Swertia tetraptera* | Gentianaceae | 0.23 | 0.01 | 0.00 | 0.03 | 0.03 | 0.75 | 0.07 | 0.35 |
| *Thalictrum alpinum* | Ranunculaceae | 0.67 | 0.35 | 0.89 | 0.84 | 0.82 | 0.28 | 0.79 | 0.03 |
| *Tibetia himalaica* | Fabaceae | 0.01 | 0.15 | 0.58 | 0.73 | 0.73 | 0.15 | 0.47 | 0.26 |
| *Tongoloa elata* | Apiaceae | 0.01 | 0.01 | 0.00 | 0.02 | 0.02 | 0.07 | 0.03 | 0.63 |
| *Viola pseudo-bambusetorum* | Violaceae | 0.41 | 0.00 | 0.23 | 0.57 | 0.13 | 0.13 | 0.00 | 0.02 |
