# Supplementary material for: Effect of Nitrogen Addition on Selection of Germination Trait in an Alpine Meadow on the Tibet Plateau
Source: Front Plant Sci. 2021 May 14;12:634850. doi: 10.3389/fpls.2021.634850 (PMC8160428; doi:10.3389/fpls.2021.634850)
Supplement: Supplementary Appendix 2 — Relative abundance of species along the nitrogen fertilization gradient. RaN0, RaN1, RaN2 and RaN3: relative abundance of species (Ra) at positions along the gradient where soil received an application of 0 (N0), 5 (N1), 10 (N2) and 20 (N3) g N m–2y–1. [file Data_Sheet_2.doc]

**Appendix S2. Relative abundance of species along the fertilization gradient. RaN0, RaN1, RaN2 and RaN3: relative abundance of species (Ra) at positions along the gradient where soil received an application of 0 (N0), 5 (N1), 10 (N2) and 20 (N3) g N m^-2^y^-1^.**

| **species** | **family** | **RaN0** | **RaN1** | **RaN2** | **RaN3** |
| --- | --- | --- | --- | --- | --- |
| *Adenophora himalayana* | Campanulaceae | 0.0000 | 0.0000 | 0.0011 | 0.0000 |
| *Agrostis hugoniana* | Gramineae | 0.0000 | 0.0024 | 0.0012 | 0.0139 |
| *Agrostis trinii* | Gramineae | 0.0000 | 0.0000 | 0.0011 | 0.0024 |
| *Ajuga lupulina* | Labiatae | 0.0000 | 0.0000 | 0.0000 | 0.0000 |
| *Allium sikkimense* | Liliaceae | 0.0071 | 0.0190 | 0.0209 | 0.0033 |
| *Anemone obtusiloba* | Ranunculaceae | 0.0167 | 0.0149 | 0.0110 | 0.0055 |
| *Anemone rivularis* | Ranunculaceae | 0.0379 | 0.0548 | 0.0503 | 0.0382 |
| *Anemone trullifolia* | Ranunculaceae | 0.0376 | 0.0057 | 0.0249 | 0.0109 |
| *Angelica nitida* | Apiaceae | 0.0000 | 0.0000 | 0.0012 | 0.0000 |
| *Artemisia tangutica* | Asteraceae | 0.0014 | 0.0000 | 0.0000 | 0.0000 |
| *Artemisia mongolica* | Asteraceae | 0.0000 | 0.0025 | 0.0000 | 0.0000 |
| *Aster diplostephioides* | Asteraceae | 0.0000 | 0.0000 | 0.0000 | 0.0000 |
| *Aster souliei* | Asteraceae | 0.0285 | 0.0328 | 0.0207 | 0.0437 |
| *Astragalus ploycladus* | Fabaceae | 0.0170 | 0.0118 | 0.0103 | 0.0013 |
| *Carex crebra* | Cyperaceae | 0.0223 | 0.0012 | 0.0024 | 0.0058 |
| *Carum buriaticum* | Apiaceae | 0.0009 | 0.0037 | 0.0024 | 0.0057 |
| *Cerastium fontanum* | Caryophyllaceae | 0.0082 | 0.0013 | 0.0050 | 0.0067 |
| *Delphinium kamaonense* | Ranunculaceae | 0.0178 | 0.0153 | 0.0217 | 0.0189 |
| *Elymus nutans* | Gramineae | 0.0652 | 0.2050 | 0.2125 | 0.3385 |
| *Euphrasia regelii* | Orobanchaceae | 0.0444 | 0.0262 | 0.0175 | 0.0000 |
| *Festuca ovina* | Poaceae | 0.0444 | 0.0128 | 0.0173 | 0.0064 |
| *Galium verum* | Rubiaceae | 0.0096 | 0.0000 | 0.0043 | 0.0078 |
| *Gentiana aristata* | Gentianaceae | 0.0000 | 0.0000 | 0.0000 | 0.0000 |
| *Gentiana farreri* | Gentianaceae | 0.0028 | 0.0044 | 0.0039 | 0.0012 |
| *Gentiana straminea* | Gentianaceae | 0.0031 | 0.0000 | 0.0000 | 0.0000 |
| *Gentianopsis paludosa* | Gentianaceae | 0.0095 | 0.0000 | 0.0000 | 0.0000 |
| *Geranium pylzowianum* | Geraniaceae | 0.0126 | 0.0179 | 0.0223 | 0.0087 |
| *Gueldenstaedtia verna* | Fabaceae | 0.0089 | 0.0184 | 0.0162 | 0.0241 |
| *Ixeris polycephala* | Asteraceae | 0.0023 | 0.0012 | 0.0112 | 0.0051 |
| *Kobresia graminifolia* | Cyperaceae | 0.1146 | 0.1502 | 0.1022 | 0.0434 |
| *Kobresia tibetica* | Cyperaceae | 0.0187 | 0.0000 | 0.0000 | 0.0000 |
| *Koeleria litvinowii* | Poaceae | 0.0111 | 0.0124 | 0.0076 | 0.0013 |
| *Leontopodium calocephalum* | Asteraceae | 0.0044 | 0.0000 | 0.0000 | 0.0000 |
| *Leontopodium souliei* | Asteraceae | 0.0000 | 0.0000 | 0.0000 | 0.0000 |
| *Ligularia virgaurea* | Asteraceae | 0.0296 | 0.0458 | 0.0436 | 0.1961 |
| *Lomatogonium carinthiacum* | Gentianaceae | 0.0044 | 0.0012 | 0.0000 | 0.0000 |
| *Morina chinensis* | Dipsacaceae | 0.0012 | 0.0000 | 0.0000 | 0.0000 |
| *Oxytropis kansuensis* | Fabaceae | 0.0066 | 0.0012 | 0.0000 | 0.0023 |
| *Parnassia trinervis* | Celastraceae | 0.0066 | 0.0023 | 0.0000 | 0.0000 |
| *Pedicularis cranolopha* | Scrophulariaceae | 0.0000 | 0.0000 | 0.0013 | 0.0000 |
| *Pedicularis kansuensis* | Orobanchaceae | 0.0089 | 0.0012 | 0.0000 | 0.0000 |
| *Plantago asiatica* | Plantaginaceae | 0.0030 | 0.0058 | 0.0059 | 0.0013 |
| *Plantago depressa* | Plantaginaceae | 0.0000 | 0.0012 | 0.0000 | 0.0000 |
| *Pleurospermum hookeri* | Apiaceae | 0.0000 | 0.0026 | 0.0038 | 0.0000 |
| *Poa crymophila* | Poaceae | 0.0014 | 0.0049 | 0.0206 | 0.0214 |
| *Poa pachyantha* | Poaceae | 0.0164 | 0.0193 | 0.0581 | 0.0634 |
| *Potentilla anserina* | Rosaceae | 0.0000 | 0.0000 | 0.0000 | 0.0000 |
| *Potentilla saundersiana* | Rosaceae | 0.0697 | 0.0516 | 0.0216 | 0.0241 |
| *Ranunculus tanguticus* | Ranunculaceae | 0.0238 | 0.0179 | 0.0226 | 0.0182 |
| *Rumex acetosa* | Polygonaceae | 0.0007 | 0.0011 | 0.0045 | 0.0000 |
| *Saussurea stella* | Asteraceae | 0.0099 | 0.0207 | 0.0096 | 0.0234 |
| *Saussurea hieracioides* | Asteraceae | 0.0000 | 0.0000 | 0.0023 | 0.0000 |
| *Saussurea nigrescens* | Asteraceae | 0.1210 | 0.0595 | 0.0874 | 0.0868 |
| *Saussurea pachyneura* | Asteraceae | 0.0000 | 0.0036 | 0.0013 | 0.0000 |
| *Scirpus distigmaticus* | Cyperaceae | 0.0283 | 0.0056 | 0.0013 | 0.0032 |
| *Scutellaria baicalensis* | Lamiaceae | 0.0000 | 0.0428 | 0.0011 | 0.0000 |
| *Stellaria uda* | Caryophyllaceae | 0.0016 | 0.0025 | 0.0038 | 0.0147 |
| *Stipa aliena* | Asteraceae | 0.0150 | 0.0249 | 0.0228 | 0.0072 |
| *Swertia tetraptera* | Gentianaceae | 0.0000 | 0.0000 | 0.0013 | 0.0000 |
| *Thalictrum alpinum* | Ranunculaceae | 0.0297 | 0.0158 | 0.0261 | 0.0293 |
| *Tibetia himalaica* | Fabaceae | 0.0315 | 0.0056 | 0.0033 | 0.0000 |
| *Tongoloa elata* | Apiaceae | 0.0083 | 0.0210 | 0.0178 | 0.0157 |
| *Viola pseudo-bambusetorum* | Violaceae | 0.0000 | 0.0000 | 0.0000 | 0.0000 |
