# Supplementary material for: Effect of Nitrogen Addition on Selection of Germination Trait in an Alpine Meadow on the Tibet Plateau
Source: Front Plant Sci. 2021 May 14;12:634850. doi: 10.3389/fpls.2021.634850 (PMC8160428; doi:10.3389/fpls.2021.634850)
Supplement: Supplementary Appendix 3 — Germination trait diversity indices (functional richness, FRic; functional evenness, FEve; and functional divergence, FDiv) at different levels of nitrogen addition along the nitrogen fertilization gradient. [file Data_Sheet_3.doc]

**Appendix S3. Germination trait diversity indices (functional richness, FRic; functional evenness, FEve; and functional divergence, FDiv) at different levels of nitrogen addition along the fertilization gradient.**

| **Plot** | **N0** | | | **N1** | | | **N2** | | | **N3** | | |
| --- | --- | --- | --- | --- | --- | --- | --- | --- | --- | --- | --- | --- |
|  | FRic | FEve | FDiv | FRic | FEve | FDiv | FRic | FEve | FDiv | FRic | FEve | FDiv |
| 1 | 4.6889 | 0.5868 | 0.8730 | 5.6596 | 0.7043 | 0.8332 | 4.3516 | 0.6642 | 0.8503 | 3.0975 | 0.6855 | 0.8849 |
| 2 | 6.3525 | 0.6135 | 0.7832 | 2.2165 | 0.6255 | 0.7852 | 4.4111 | 0.6723 | 0.8839 | 1.1970 | 0.5531 | 0.8738 |
| 3 | 3.0489 | 0.6471 | 0.7912 | 3.3848 | 0.6218 | 0.9031 | 4.7354 | 0.6201 | 0.8535 | 0.7083 | 0.7097 | 0.8866 |
| 4 | 4.7348 | 0.6188 | 0.7987 | 3.2909 | 0.6883 | 0.8829 | 1.7550 | 0.6229 | 0.8027 | 0.4088 | 0.5886 | 0.8982 |
| 5 | 4.4519 | 0.6514 | 0.7667 | 2.9752 | 0.5740 | 0.8459 | 1.7889 | 0.6627 | 0.9007 | 1.8743 | 0.5616 | 0.7220 |
| Mean | 4.6554 | 0.6235 | 0.8026 | 3.5054 | 0.6428 | 0.8501 | 3.4084 | 0.6484 | 0.8582 | 1.4572 | 0.6197 | 0.8531 |
